# Near infrared-emitting multimodal nanosystem for in vitro magnetic hyperthermia of hepatocellular carcinoma and dual imaging of in vivo liver fibrosis

**DOI:** 10.1038/s41598-023-40143-3

**Published:** 2023-08-09

**Authors:** Shaiju S. Nazeer, Ariya Saraswathy, Nirmala Nimi, Hema Santhakumar, Parvathy Radhakrishnapillai Suma, Sachin J. Shenoy, Ramapurath S. Jayasree

**Affiliations:** 1 Department of Chemistry, Indian Institute of Space Sciences and Technology, Thiruvananthapuram, 695547 Kerala India; 2 Division of Biophotonics and Imaging, Biomedical Technology Wing, Sree Chitra Tirunal Institute for Medical Sciences & Technology, Poojappura, Thiruvananthapuram, 695 012 Kerala India; 3 Department of Physics, HHMSPBNSS College, Thiruvananthapuram, 695 040 Kerala India; 4 Division of In Vivo Models and Testing, Biomedical Technology Wing, Sree Chitra Tirunal Institute for Medical Sciences & Technology, Poojappura, Thiruvananthapuram, 695 012 Kerala India

**Keywords:** Materials science, Nanoscience and technology, Optics and photonics

## Abstract

Prolonged usage of traditional nanomaterials in the biological field has posed several short- and long-term toxicity issues. Over the past few years, smart nanomaterials (SNs) with controlled physical, chemical, and biological features have been synthesized in an effort to allay these challenges. The current study seeks to develop theranostic SNs based on iron oxide to enable simultaneous magnetic hyperthermia and magnetic resonance imaging (MRI), for chronic liver damage like liver fibrosis which is a major risk factor for hepatocellular carcinoma. To accomplish this, superparamagnetic iron oxide nanoparticles (SPIONs) were prepared, coated with a biocompatible and naturally occurring polysaccharide, alginate. The resultant material, ASPIONs were evaluated in terms of physicochemical, magnetic and biological properties. A hydrodynamic diameter of 40 nm and a transverse proton relaxation rate of 117.84 mM^−1^ s^−1^ pronounces the use of ASPIONs as an efficient MRI contrast agent. In the presence of alternating current of 300 A, ASPIONs could elevate the temperature to 45 °C or more, with the possibility of hyperthermia based therapeutic approach. Magnetic therapeutic and imaging potential of ASPIONs were further evaluated respectively in vitro and in vivo in HepG2 carcinoma cells and animal models of liver fibrosis, respectively. Finally, to introduce dual imaging capability along with magnetic properties, ASPIONs were conjugated with near infrared (NIR) dye Atto 700 and evaluated its optical imaging efficiency in animal model of liver fibrosis. Histological analysis further confirmed the liver targeting efficacy of the developed SNs for Magnetic theranostics and optical imaging as well as proved its short-term safety, in vivo.

## Introduction

In the last two decades, advances in nanomedicine have vastly improved the diagnosis and therapy of various diseases. However, repeated exposure to these nanomaterials raises more challenges of systemic toxicity, biocompatibility, less photostability and non-targeted distribution inside the body. Smart nanomaterials are superior to traditional nanosystems because they have regulated physical, chemical, and biological properties. Based on the stimulus required to trigger the response, SNs can be classified into physical, thermal, electrical/electrochemical, optical, magnetic, pH/redox/enzyme responsive nanomaterials^1,2^. Due to their unique characteristics, SNs are excellent choice for several applications, including biosensors, controlled drug release, and the treatment of numerous diseases. Smart nanomaterials employed in cancer diagnostics and imaging respond only to the tumour microenvironment where they ‘turn on’, while they remain inert in normal cells, reducing the side-effects^1–4^.

Among smart nanomaterials, magneto-responsive smart nanomaterials (MRSNs), which respond to an applied magnetic field, have excellent prospects for both diagnosis and therapy^3^. MRSNs can improve the field of theranostics and associated domains like magnetic separation, cellular labelling, immunoassays, MRI, magneto-sensors, and treatment-specific modalities like magnetic hyperthermia and magnetic-guided drug delivery. Suitable surface morphology, charge, or surface modification of MRSNs are useful for binding with biopolymers, nucleic acids, or optical dyes to facilitate multifunctional properties and targetability^3,5–10^.

Fatty liver is the earliest indication of many of the liver diseases, of which majority are believed to be the effect of factors like chronic infection with hepatitis B or C virus, heavy alcohol consumption, obesity, type 2 diabetes, smoking and after effects of aflatoxin-contaminated foods^11–13^. Fatty liver with hepatic inflammation may initiate with symptoms of fibrosis and progress to cirrhosis and finally lead to HCC in many cases, which is the leading sub type of primary liver cancer^14–16^. However, the disease is manageable and reversible by timely diagnosis of fibrosis and by proper therapeutic intervention at this stage^17,18^. Thus, the importance of early and accurate diagnosis and proper treatment is undoubtedly exemplified.

Though MRI is the best available in vivo diagnostic technique currently used for soft tissue imaging including cancerous transformations, it often requires external contrast agents for better visibility^19–22^. In the case of fatty liver, the accumulated fat has low response to conventional gadolinium-based contrast agents which is predominantly a positive contrast agent due to its prominent longitudinal relaxation (T_1_) property. So, contrast agents with transverse relaxation (T_2_) property that can provide T_2_ signal are preferred for liver imaging. Super paramagnetic metallic nanoparticles with proper surface coating are known for their competence for liver imaging^23,24^. Furthermore, they have additional thermal property in the presence of alternating current, to induce cell death, so that it can have the dual functions of diagnosis and therapy^6,25,26^.

In this study, we have developed superparamagnetic iron oxide nanoparticles (SPIONs) based multifunctional theranostic agent for liver associated diseases. Alginate stabilised SPIONs (ASPIONs) with average size of 40 nm, with excellent T_2_ relaxation and thermal property were developed for MR imaging and hyperthermia-based treatment. Additionally, a water-soluble dye with emission in the NIR range, Atto-700 coupled to SPIONs gave the optical imaging capability. Cyto compatibility, thermal property and imaging potential of SNs were evaluated in vitro and in vivo.

## Materials and methods

### Materials

FeCl_3_ anhydrous, FeCl_2_∙4H_2_O, NaOH, 35% HCl (All from Merck, Germany/India) and Sodium Alginate Polysaccharide (Sigma Aldrich, India) were used for the preparation of ASPIONs. NIR emitting dye (Atto 700) was procured from Sigma Aldrich, India for optical imaging. Deionised Milli Q water of resistivity 18.2 MΩ was used throughout the work. All other chemicals and reagents used were of analytical grade.

For the cell culture study, Dulbecco’s Modified Eagle’s Medium (DMEM) (Himedia Laboratories Pvt. Ltd, Mumbai, India), sodium bicarbonate, Gentamicin (Himedia, Germany), Amphotericin B solution (Sigma-Aldrich, Germany), Fetal bovine serum (FBS), antibiotic–antimycotic mix (Himedia, India) and 3-(4, 5-dimethylthiazol-2-yl)-2.5-diphenyltetrazolium bromide (MTT) were used. HepG2 carcinoma cells were supplied from National Centre for Cell Science (NCCS, Pune, India). Carbon tetrachloride (CCl_4_) purchased from Spectrum Reagents & Chemicals Ltd, India and olive oil were used to develop fibrosis in rodents.

### Synthesis

#### Synthesis of SPIONs and ASPIONs

Alkaline co-precipitation method was adopted for the preparation of SPIONs. Briefly, FeCl_3_ anhydrous and FeCl_2_∙4H_2_O were mixed in the 2:1 molar ratio in deionized water. The mixing continued until a homogenous solution was obtained. 1 M NaOH solution was added drop wise, and the mixture was maintained at 80 °C under inert atmosphere to obtain the SPIONs as the black precipitate. The same condition was maintained for another 2 h to complete the reaction. The SPIONs obtained were magnetically separated and washed by centrifugation with deionized water containing HCl.

The SPIONs were dispersed in 1.2% (w/v) sodium alginate solution and stirred overnight. The alginate coated SPIONs (ASPIONs) were washed by centrifugation to remove the alginate debris. 

#### Synthesis of ASPION-AT

1.2 mg ml^−1^ concentration of ASPIONs (10 ml) was mixed with 20 µl of 1.3 µg ml^−1^ of Atto 700 dye and sonicated for about 6 h in the dark. After sonication, a color change from pale brown to pale green was considered as an indication for the formation of Atto modified ASPIONs (ASPION-AT). The suspension was centrifuged to eliminate the unreacted dye and used for further characterization.

#### Physico chemical characterisation

The particle size of the nanoparticle ASPIONs was analysed by Dynamic Light Scattering (DLS) technique and zeta potential by Zetasizer, Nano ZS with MPT-2 auto-titrator (Malvern Instruments Limited, UK) at 25 °C. The size and shape of the nanoparticles were examined by transmission electron microscopy at 100 kV (TEM, JEM-2010, JEOL, Tokyo, Japan). Samples were prepared by depositing a few droplets of dilute nanoparticles solution on to a formvar-coated copper grid.

The surface stabilisation and the structure of ASPIONs were studied using FTIR spectrometer (Thermo Nicolet 5700 FTIR spectrometer (USA)) using KBr pellet method. The structure and phase analysis of ASPIONs was analysed by means of X’Pert PRO X-ray diffraction (XRD) instrument using Cu Kα radiation of 1.5406 Å at 40 kV and a 20 mA current. The crystal structure of the nanoparticles was assessed by evaluating the peak position and intensities at the diffraction angle range, 2θ—10° to 80°.

The thermal decomposition property of ASPION nanoparticles was studied using Thermo gravimetric analysis (TGA), SDT Q600 (simultaneous TGA-DTA, TA Instruments, USA) instrument. The lyophilised sample was analysed within a temperature range from 25 to 800 °C at a ramp rate of 10 °C min^−1^ under nitrogen atmosphere. The amount of alginate present in the ASPIONs was evaluated by the percentage decomposition at different temperatures and compared with that of bare alginate.

#### Magnetic characterisation

Lakeshore model 7410 vibrating sample magnetometer (VSM) was used to measure magnetic hysteresis of ASPIONs. A maximum magnetic field of 150 Oe was applied to obtain the measurement. The superparmagnetic nature and the saturation magnetization (Ms) of the ASPIONs was evaluated using the Magnetisation (M) versus magnetic field strength (H) plot.

Magnetic relaxivity measurements of ASPIONs were carried out using a 1.5 T whole body MRI scanner (MAGNETOM Avento Tim, Siemens, Munich, Germany) with a 12-channel radio frequency coil. Homogenous aqueous phantoms of various Fe concentrations ranging from 0 to 0.45 mM were scanned under the MRI machine. By applying an inversion recovery MRI sequence, longitudinal relaxation time, T_1_ of the samples were measured. The repetition time (TR) and echo time (TE) were set at 4000 and 11 ms respectively and the MR signal was measured by changing the inversion time (TI) from 50 to 3000 ms. Transverse relaxation time, T_2_ was carried out using a modified T_2_ relaxometry spin echo sequence. At a fixed TR of 2000 ms and by varying TE from 15 to 120 ms (step size—15 ms) MR signal variation was measured. Using the resultant image pixel intensity maps corresponding to each concentration, longitudinal and transverse relaxation rates (r_1_ and r_2_) of ASPIONs were calculated via linear regression analysis by plotting relaxation rates against iron concentration.

The Magnetic hyperthermia efficiency of ASPIONs was examined using a laboratory induction system (Ambrell EASYHEAT, Rochester, USA) having a magnetic field frequency of 275 kHz. For heat generation, a solenoid coil with dimensions, 4 × 2.6 cm (diameter and length) with a total number of 6 turns was set as the sample compartment. An alternating magnetic field (AMF) was applied to the sample. The variation in temperature of the nanoparticle in the aqueous suspension with respect to time was measured. At a fixed frequency of 275 kHz, current was altered from 200 to 400 A in steps of 50 A. By keeping a 1.5 ml non-magnetic vial with 1 ml of sample in distilled water suspension at the centre of the coil, induction heating treatment was carried out. Using a noncontact mode IR thermometer (Fluke 572, Germany), temperature profile was recorded at every 1 min. Specific loss power (SLP) of developed ASPIONs was evaluated using the following equation by means of the obtained temperature profile.

$$SLP = \frac{CVs}{m}\frac{dT}{{dt}}$$

where C is the volumetric specific heat capacity of the sample (C_water_ = 4185 J L^−1^ K^−1^ C), Vs is the sample volume, m is the mass of the magnetic material present in the sample volume and dT/dt is the initial linear rise in temperature versus time dependence^27^.

#### Optical properties

UV–Vis absorption spectroscopy (Shimadzu UV Spectrophotometer-UV-1800, Japan), fluorescence spectroscopy (Fluorolog-III; JobinYvon Inc., USA) and fluorescence imaging (Xenogen IVIS Spectrum) were performed to understand the optical property of ASPION–AT nanoparticles. Excitation wavelength of Atto dye, 680 nm was used to obtain the emission spectrum. The optical imaging capability of the Atto conjugated particles was analyzed using the optical imaging system (IVIS Spectrum).

### In vitro evaluations

#### In vitro cytotoxicity and reactive oxygen species (ROS) assessment

The cytocompatibility of the particles was evaluated on hepatoma cells (HepG2) using MTT assay using standard protocols^28^.

A cell permeable probe 2′7′-dichlorodihydrofluorescein diacetate (H_2_DCFDA) was used to detect any intracellular ROS that is developed within the Hep G2 cells upon ASPION treatment. The assay was performed following instructions from the manufacturer (Invitrogen cat #C6827). The assay used 10 μM H_2_O_2_ as a positive control. ROS levels from untreated cells maintained under similar ambient conditions were used to normalize the ASPION treatment values and to minimize the background interference. The fluorescence intensity of the formed 2′,7′-dichlorofluorescein as a result of carboxy-DCFDA hydrolysis was analysed in a 96-well spectro-fluorimeter plate reader (Synergy H1 hybrid multi-mode microplate reader, Bio-Tek) at an excitation and emission wavelength of 492 and 527 nm respectively.

#### In vitro magnetic hyperthermia

For in vitro magnetic hyperthermia treatment, HepG2 cells seeded at a density of 1 × 10^4^ cells in 12 mm coverslips and incubated with 2 mg ml^−1^ of sterile ASPIONs and then subjected to alternating magnetic field of 33.8 mT and 275 kHz for 15 min. An infrared thermometer was used to monitor the temperature change in cell environment. Live/dead assay with dual staining using acridine orange and ethidium bromide was done to study the cell death induced by magnetic hyperthermia treatment. Cells without any treatment were used as control and cells incubated with ASPION alone without AMF treatment were also studied for comparison. Additionally, the cells after magnetic hyperthermia were studied using Environmental Scanning Electron Microscopy (ESEM-FEI QUANTA 200).

### In vivo imaging

#### Animal model development

Prior approval from Institutional Animal Ethics Committee of Sree Chitra Tirunal Institute for Medical Sciences and Technology was obtained for the animal studies (No: B 2982011 IX, dated: 19-10-2011). All experiments were performed in accordance with the relevant guidelines and regulations. Animal study was carried out in compliance with the ARRIVE guidelines. Liver fibrosis in rat and mice model was developed as per the reported protocol using CCl_4_-olive oil mixture^29^. Male Wistar rats (n = 6) weighing ~ 220 g were used for the in vivo MRI study. CCl_4_-olive oil mixture (1:1 ratio) was injected intra-peritoneally twice a week for 6 weeks to induce liver fibrosis in rats. Male Swiss albino mice (n = 6) weighing ~ 35 g were used for optical imaging. Here, the liver fibrosis was developed by treating 1:7 ratio of CCl_4_: olive oil mixture intra-peritoneally at the dosage of 1 μL g^−1^ body weight, every 5 days, for 4 weeks. After the stipulated time, animals with elevated liver enzymes as evaluated by the liver function test were used for in vivo imaging.

#### In vivo magnetic resonance imaging

Whole body clinical MRI scanner (1.5 T) with a head coil was used for MR imaging of rat model of fibrosis. A multisection T_2_-weighted turbo spin echo sequence (TE 125 ms; TR 5780 ms; flip angle 90; FOV 98 mm × 140 mm; slice thickness 3 mm) was used to carry out the MRI. Before administering the contrast agent, pre contrast imaging was carried out. ASPIONs was administered intravenously through tail vein at a dosage of 2.17 mg ml^−1^ (0.04 mM) Fe/kg body weight. After 15 min, post contrast images were also acquired. In order to assess signal intensity variation after contrast administration, signal intensity extraction and percentage evaluation was carried out on pre and post contrast images.

#### In vivo optical imaging

Liver fibrosis induced mice were administrated with ASPION-AT at a dosage of 0.04 mM of Fe/kg through tail vein and imaged using the Xenogen IVIS Spectrum imaging system. The excitation and emission filters corresponding to that of ATTO dye (675 and 720 nm respectively) was used. Fibrosis induced mice injected with saline was used as negative control. Ex vivo fluorescent images of the harvested organs were recorded after 2 h of injection (on sacrifice of the animals) to test for the organ specific targeting capability as well as particle distribution and clearance.

#### Histopathology

After 2 h of contrast (ASPION) injection, animals were euthanized by carbon dioxide asphyxiation method, and liver tissue was harvested from normal and fibrosis induced animals. Liver tissue was fixed with 10% neutral buffered formalin for 3 days and then embedded in paraffin and sectioned for further analysis. Pathological details of the liver tissue were evaluated using haematoxylin and eosin (H&E). Masson’s Trichrome (MT) stain enabled the collagen fibres evaluation in liver tissues and the presence of iron was evaluated using Pearls’ Prussian blue (PB) staining.

## Results and discussions

### Synthesis and characterization

Co-precipitation method, an easy and convenient way of synthesis was used for the preparation of alginate stabilized SPIONs as a potential MR contrast and hyperthermia agent. Alginate, a naturally occurring biopolymer was preferred due to its attractive features like biocompatibility, biodegradability, antibacterial activity, hydrophilicity, and nontoxicity. Moreover, the presence of amenable functional groups for better reactivity for further functionalization was added advantage, which was utilized to conjugate optical probe to use it as a multimodal imaging nanoprobe. Till date, very few reports are available on alginate functionalized magnetic nanoparticles^30–32^ and none extended the same to the development of multimodal nanoprobe for diagnosis and therapy, and has proven the concept in in vivo models.

**Figure 1:**
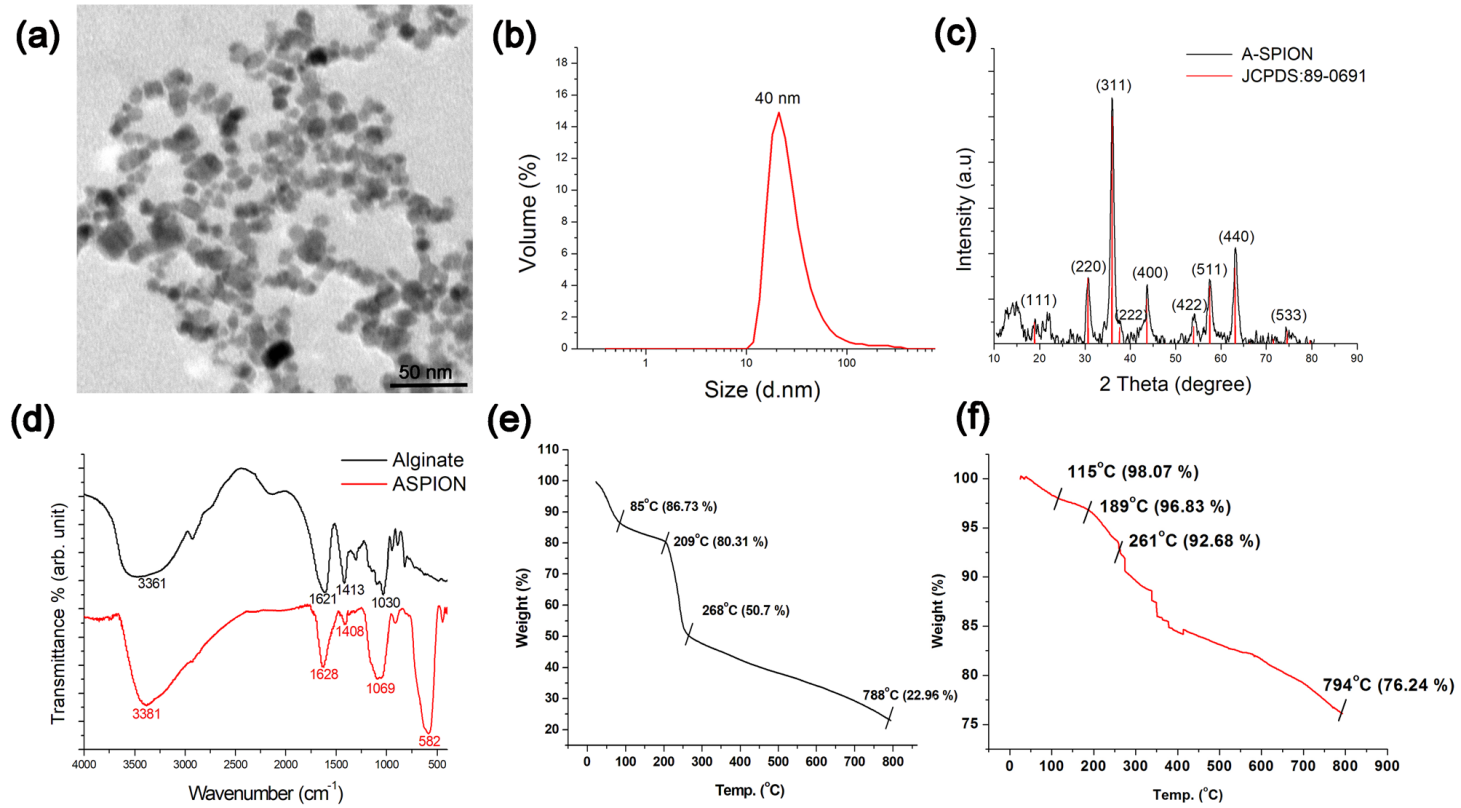
Size, shape and physico-chemical characterization of ASPION. (**a**) TEM image showing the shape and size of ASPIONs, (**b**) particle size distribution using dynamic light scattering graph, (**c**) XRD pattern of ASPION confirming the magnetite phase, (**d**) FTIR spectra of Alginate and ASPION and thermo gravimetric analysis of (**e**) alginate and (**f**) ASPION showing the thermal decomposition.

TEM micrographs showed the average size of the ASPIONs as 12 ± 3 nm without any aggregation (Fig. 1a) whereas the hydrodynamic diameter was 38 ± 4 nm (Fig. 1b).Though several factor like the concentration of alkaline medium and the precipitation temperature can significantly influence the size of the materials formed, the hydrodynamic diameter of the material of current study matches with that of some of the previously reported alginate stabilized SPIONs^31,32^. XRD pattern of ASPIONs showed the inverse spinel structure of the magnetite phase (Fig. 1c) which corresponds to the JCPDS card No. 89-0691 and the previously reported work^33^. Infrared spectrum of sodium alginate (Fig. 1d) exhibited peaks around 3361, 1621, 1413 and 1030 cm^−1^ corresponding to the O–H, C–O, C–H and C–C vibrations. Shifts in the O–H, C–O and C–C peaks along with the undisturbed C–H vibration mode of alginate and the presence of Fe–O peak at 582 cm^−1^ confirm the formation of ASPIONs. TGA curve of Alginate (Fig. 1e) shows decomposition at 85 °C followed by degradation at 209 °C and 268 °C with a 50% weight loss^34^. At the temperature around 788 °C, a total weight loss of 77% was observed. The corresponding degradation of alginate in ASPIONs was around 115 °C, 189 °C, 261 °C and 794 °C with a total weight loss of 24% (Fig. 1f). The degradation temperature of ASPIONs got shifted to a lower temperature on comparison with that of the stabilizer, sodium alginate indicating an early decomposition of ASPIONs with temperature. Catalytic behavior of iron oxide nanoparticles is attributed to the observed shift in the decomposition temperature and the fast decomposition.

### Magnetic and thermal properties

**Figure 2:**
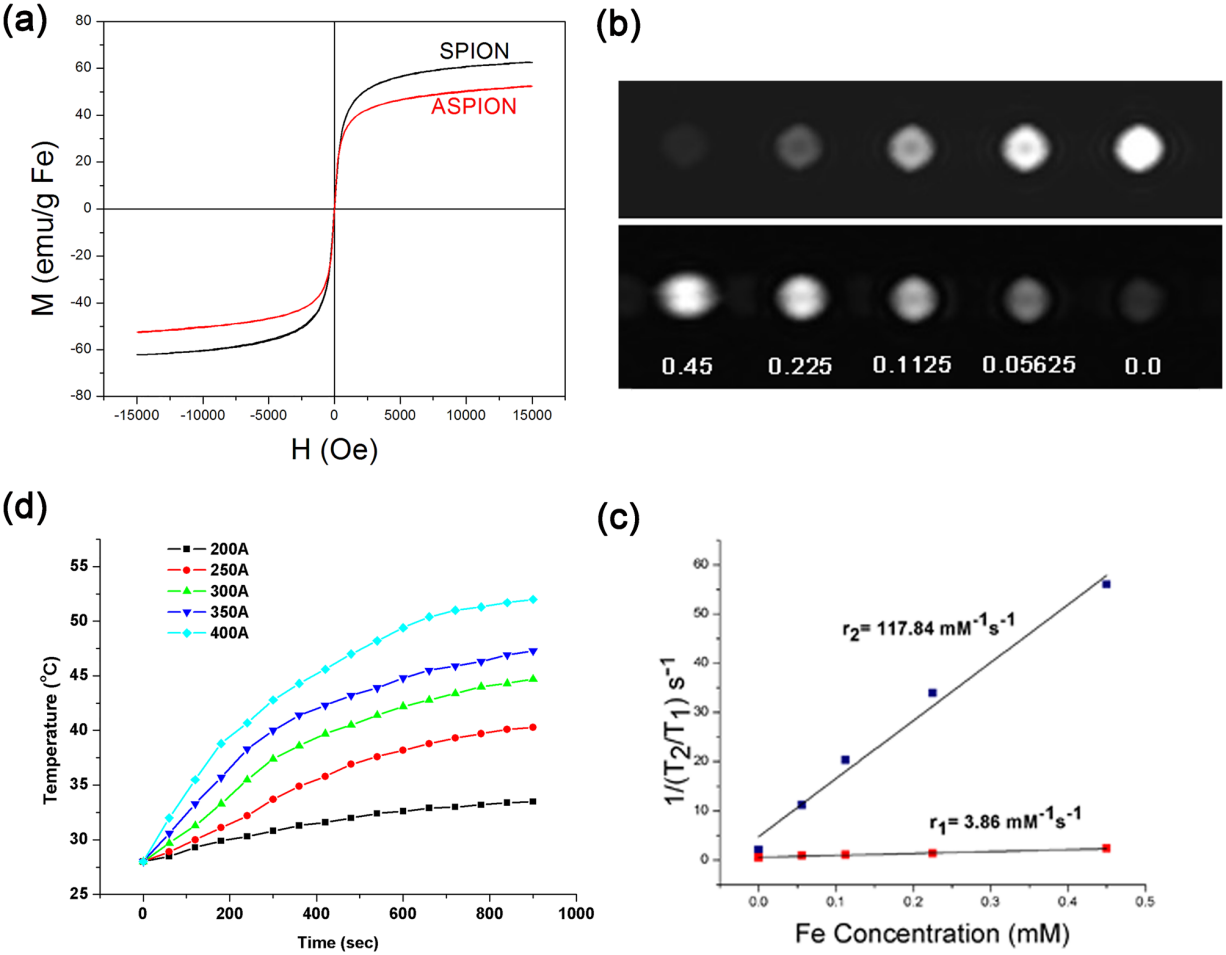
Magnetic property of NPs. (**a**) Magnetic hysteresis curves of SPION and ASPION showing characteristics of superparamagnetic nature. (**b**) Longitudinal and transverse relaxation images of ASPION in aqueous solution with varying concentrations (0–0.45 mM) (**c**) corresponding r_1_ and r_2_ relaxation rate of ASPIONs. (**d**) hyperthermia effect of ASPION under alternating magnetic field at current ranging from 200 to 400 A.

The magnetic nature and saturation magnetization of the ASPIONs was studied using vibrational sample magnetometry. Both SPIONs and ASPIONs showed the characteristic hysteresis pattern of superparamagnetic materials at room temperature, showing zero coercivity (Fig. 2a) and good magnetic saturation. The observed pattern of the hysteresis curve is an indication of the particle’s single domain existence with only one orientation of magnetic moment. Here, the magnetization reduces from plateau region to zero on removal of the magnetic field. This further supports the magnetite phase of the developed material^35^. This is an essential property for the nanoprobe to work as a suitable T_2_ contrast agent. SPIONs showed high saturation magnetization of 62.7 emu g^−1^ whereas surface modification with alginate caused a slight decrease in saturation magnetization (53 emu g^−1^)^36^. It is also reported that superparamagnetic property helps in the uniform dispersion of the particles in solution without any aggregation^37^. Hence superparamagnetic property, which is highly influenced by the size of the particles, is important for smooth circulation of the material through the blood for its use as MRI contrast agent.

Based on the pixel intensity expressions of the MRI images of the colloidal phantom of the material, longitudinal and transverse relaxation rates r_1_ and r_2_ of ASPIONs were calculated. Transverse relaxivity rate was calculated from the slope of linear plot of 1/T_2_ versus iron concentration. An r_2_ value of 117.84 mM^−1^ s^−1^ was exhibited by ASPIONs (Fig. 2b). Longitudinal relaxivity rate value calculated by the linear fit has a value of 3.86 mM^−1^ s^−1^ for r_1_. The increase in T_2_ relaxivity compared to bare SPIONs^36^ depends mainly on the effect of the delay in relaxation of the protons bound to the alginate coated SPION and the water protons^38^. Compared to other iron oxide based contrast agents of similar sizes, r_2_ of 117.84 mM^−1^ s^−1^ and relaxivity ratio (r_2_/r_1_) of 30.53 obtained for ASPIONs are high and hence is more suitable to use it as an efficient T_2_ contrast agent for MRI^39–41^.

ASPIONs were also evaluated for its heat generating capability for cancer therapy and the results are shown in Fig. 2c. A concentration of 5 mg ml^−1^ of ASPIONs raised the temperature to more than 40–55 °C on the application of electric current varying from 200-400 A with a constant frequency of 275 kHz for 8 min, establishing it as an apt contender for hyperthermia. For the temperature based therapy, temperatures of the order of 40–43 °C can favor efficient cancer cell death without destructing the surrounding normal cells^42–45^. The temperature rise could be optimized by adjusting the current. Here, the temperature was highest for the maximum current of 400 A. Evaluation of specific loss power (SLP) of a material can act as an indicator for the magnetic hyperthermia efficacy. SLP is defined as the amount of energy converted into heat per time and mass. It is reported that SLP critically depend on the diameter of Fe_3_O_4_ nanoparticles, having a maximum at diameters ~ 16–17 nm. SLP also depends slightly on the frequency of the applied AMF (in the range 100–500 kHz) which is directly proportional to the diameter of the nanoparticle in the range 13–20 nm. For nanoparticles having size above or below this range of diameter, SLP rapidly decreases to zero, and hyperthermia effect will not be achieved. Also, the SLP increases approximately in proportion to the increase in AMF frequency^46^.

Therapeutic efficacy of ASPIONs was calculated based on SLP and is given in Table 1. As the diameter of ASPIONs falls within the critical diameter and the frequency of the AMF chosen was also suitable for effective hyperthermia, ASPIONs enhanced the temperature and saturations on increasing the current of the AMF from 200 to 400 A.

**Table 1:** Specific loss power values obtained for ASPIONs for varying current and magnetic field strength.

Sl no	Current through coil (A)	Magnetic field strength (mT)	SLP
			ASPION 5 mg ml^−1^
1	200	14.4	9.79
2	250	19.33	20.09
3	300	24.166	29.04
4	350	28.99	40.18
5	400	33.83	50.64

### Optical properties

Multimodal nanoprobe with both magnetic and optical properties was prepared by incorporating NIR emitting dye, Atto 700 with ASPION by physical interaction. Atto 700 is a zwitterionic dye with high fluorescence quantum yield, good water solubility and high photostability and is a strong electron acceptor. Simple electrostatic interaction of the hydroxyl groups of ASPIONs and COO^–^ group of Atto 700 dye resulted in the strong physical interaction between the two and formed a very stable complex, ASPION-AT.

**Figure 3:**
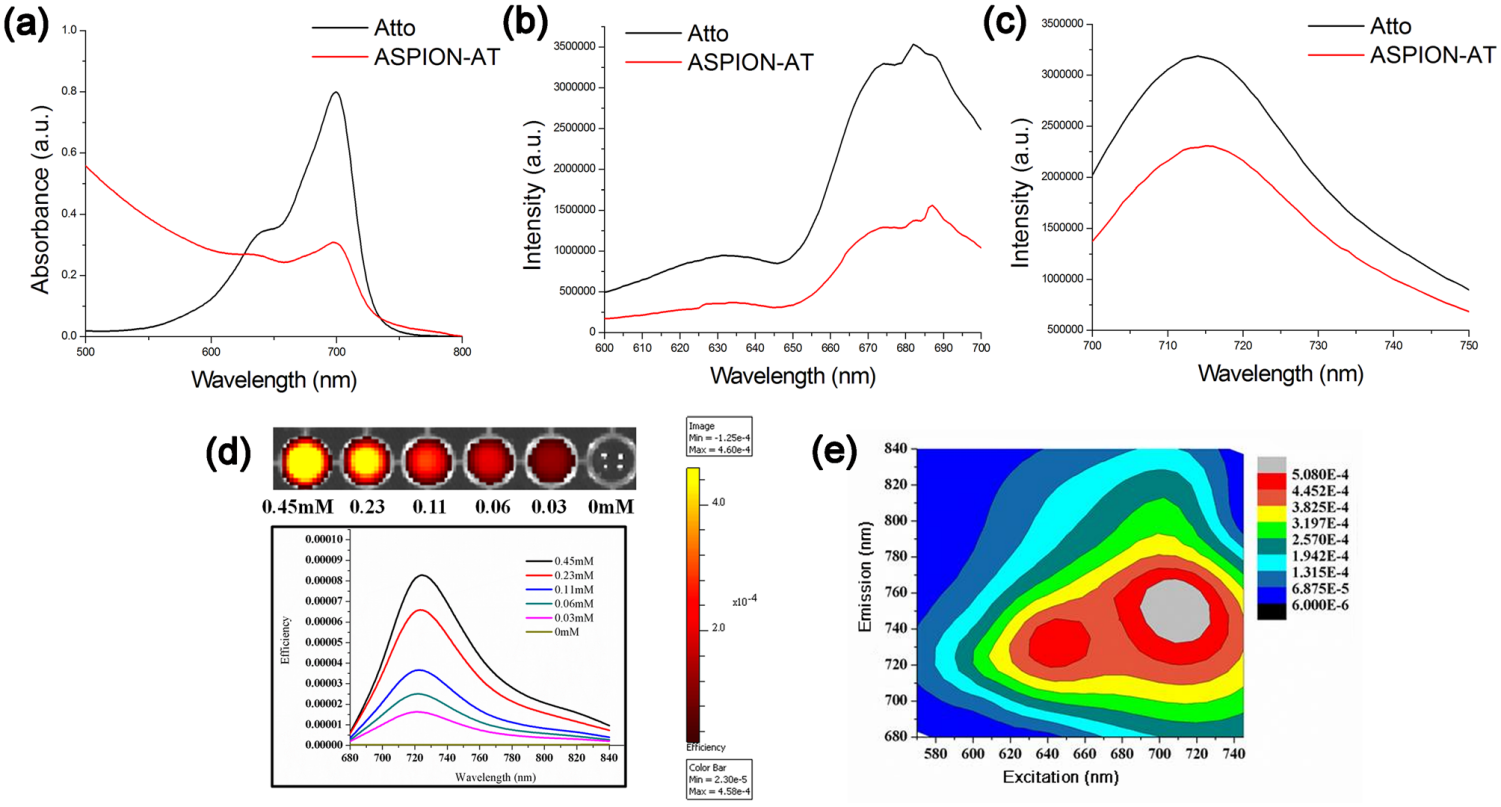
Optical properties of developed SNs (**a**) UV–visible absorption, (**b**) excitation and (**c**) emission spectra of Atto and ASPION-AT. (**d**) Fluorescence images of different concentrations of ASPION-AT (above) and the corresponding spectra excited at 675 nm with emission at 710 nm. (**e**) Excitation–Emission contour plot of ASPION-AT. The colour bar on the right gives an indication about the efficiency of the probe.

The UV–Vis spectra of ASPION-AT showed an absorption maximum around 700 nm (Fig. 3a). The fluorescence excitation spectrum of ASPIONs-AT showed an excitation maximum at 680 nm and emission spectrum showed an emission maximum at 712 nm (Fig. 3b, c). The concentration dependent optical imaging efficiency of ASPION-AT using an optical imaging system is shown in Fig. 3d. Concentrations of the order of 0.03 mM dye provided good fluorescence intensity. The excitation-emission efficiency contour plot for the corresponding excitation–emission imaging is also shown in Fig. 3e. The contour plots showed two emission maxima at around 640 nm and 710 nm with the one at 710 nm being the most intense one as is seen by the intensity indicators in Fig. 3e. Atto 700 dye was chosen for its NIR emission property as this will avoid interference from the tissue autofluorescence from the visible region during the in vivo application. The excitation emission maxima at 680 nm and 712 nm overruled the interference of autofluorescence from the animal body^47–49^. Fluorescence imaging of ASPION-AT showed remarkable fluorescence efficiency that makes it suitable for the in vivo application.

### Biocompatibility study

**Figure 4:**
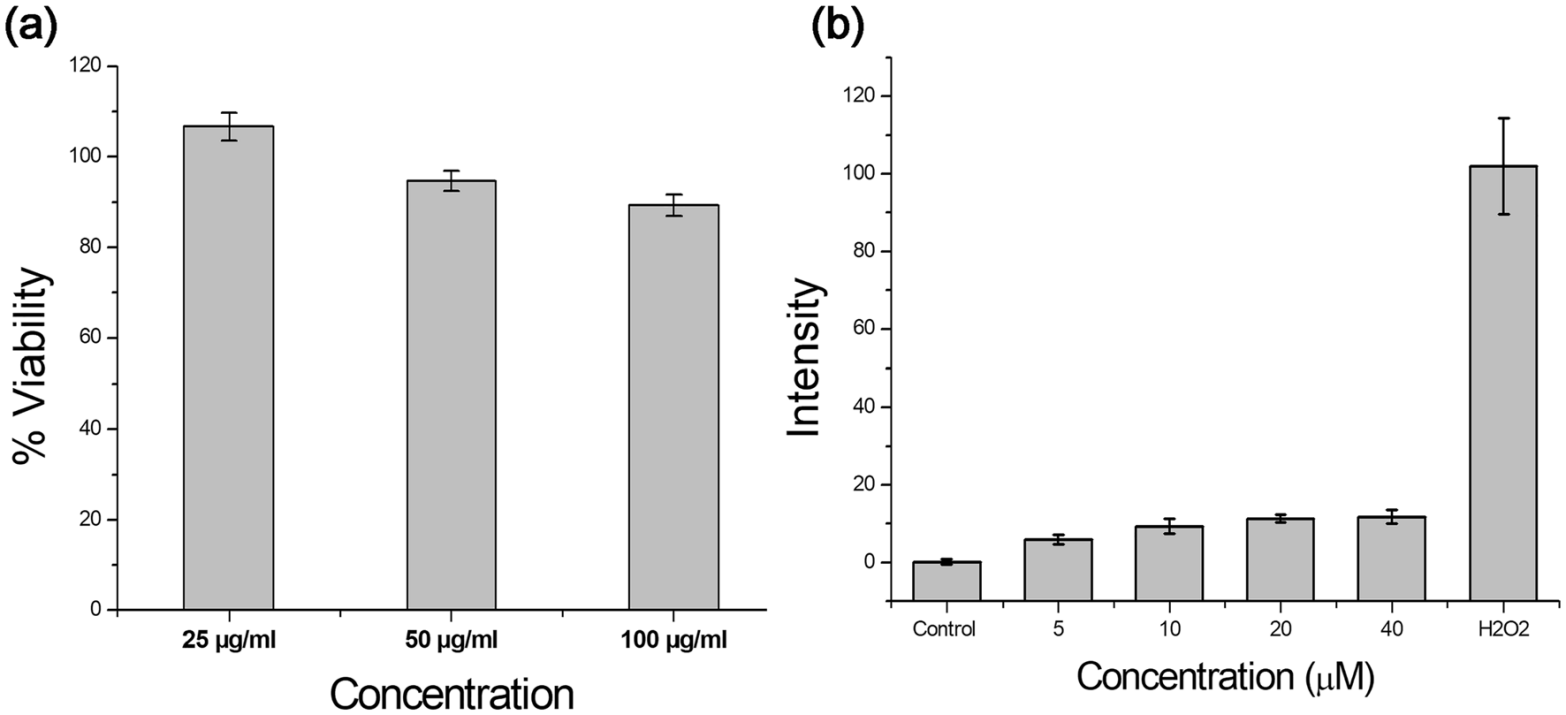
(**a**) Cytotoxicity evaluation of ASPIONs using different concentrations (25–100 μg ml^−1^). (**b**) Quantification of reactive oxygen species generated from ASPIONs evaluated by DCFDA assay.

Cytotoxicity of ASPIONs was assessed on hepatocellular carcinoma cells, HepG2 using MTT assay (Fig. 4a). Cell viability of 89, 94 and 106% were obtained respectively for ASPION concentrations 100, 50 and 25 µg ml^−1^. In MTT assay, total cell activity is measured on a normalized manner which is an indirect representation of cell viability. It is quite usual to increase the total cell activity if the cell number is more with respect to previous count. This accounts for the observed cell viability of 106% in the lower concentration level of the material.

A consistent redox balance is always maintained to sustain a basal level of intracellular ROS at any point of time. Nanomaterial treatment in any cells, evoke the intracellular defence machinery to overcome any adverse reactions and mark increase in the ROS production. Any intracellular ROS that is developed within the HepG2 cells upon ASPION treatment was evaluated using H_2_DCFDA. The results demonstrated only basal level of fluorescence signals arising from the ROS generated, from all the treatment concentrations even up to 40 μM of ASPIONs post 24-h treatment (Fig. 4b). This result clearly indicates that there was no significant alteration in the intracellular redox balance, post ASPION treatment which is a good indicator for the application of ASPIONs for theranostic applications.

### In vitro hyperthermia

**Figure 5:**
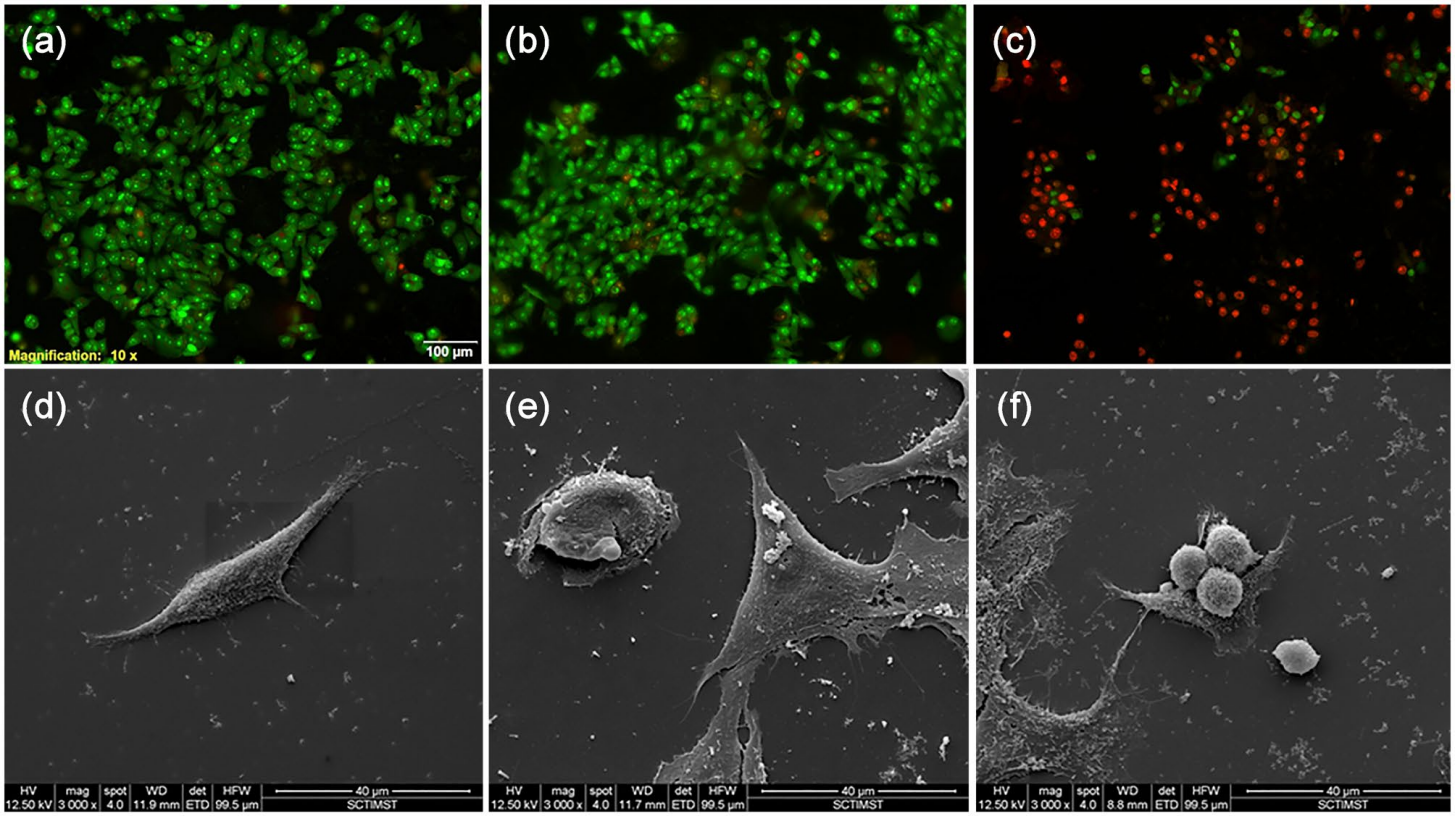
In vitro hyperthermia using ASPIONs. (**a**–**c**) Live-dead cell assay and (**d–f**) ESEM images of HepG2 cells. (**a** & **d**) Controls, (**b** & **e**) with ASPION treatment without exposure to AMF and (**c** & **f**) with ASPION treatment and exposure to AMF.

Application of controlled temperature on cancerous cells can lead to apoptotic cell death^25^. Therefore apoptosis or programmed cell death via magnetic hyperthermia is a suitable methodology to annihilate cancer cells with nominal side effects^50,51^. In this study, we have evaluated hyperthermia potential of ASPIONs on hepatocellular carcinoma cells. For this, HepG2 cells were incubated with ASPIONs for 4 h and the cells were exposed to AMF for 15 min. Cells without any treatment served as control and cells treated with ASPIONs alone without exposing to AMF was used to evaluate material toxicity associated cell death. It is evident from the live/dead assay that control cells and cells incubated with ASPIONs alone without AMF exposure showed predominantly green signal which is the characteristic of live cells in live/dead assay (Fig. 5a, b). Whereas, most of the ASPION incubated cells exposed to AMF were positively stained for apoptotic cell death (Fig. 5c).

Further, the morphological changes of cancer cells by ASPION induced apoptosis by the magnetic hyperthermia was studied using ESEM. Control cells and cells incubated with ASPIONs alone without AMF exposure retained their normal morphology under ESEM (Fig. 5d, e). However, cells incubated with ASPIONs and exposed to AMF showed the characteristics of apoptosis like membrane blebbing, loss of structural integrity, cell shrinkage and rupture of cell membrane (Fig. 5f). This indicates that ASPIONs are effective candidates for magnetic hyperthermia-based cancer therapy.

### In vivo Magnetic Resonance Imaging

**Figure 6:**
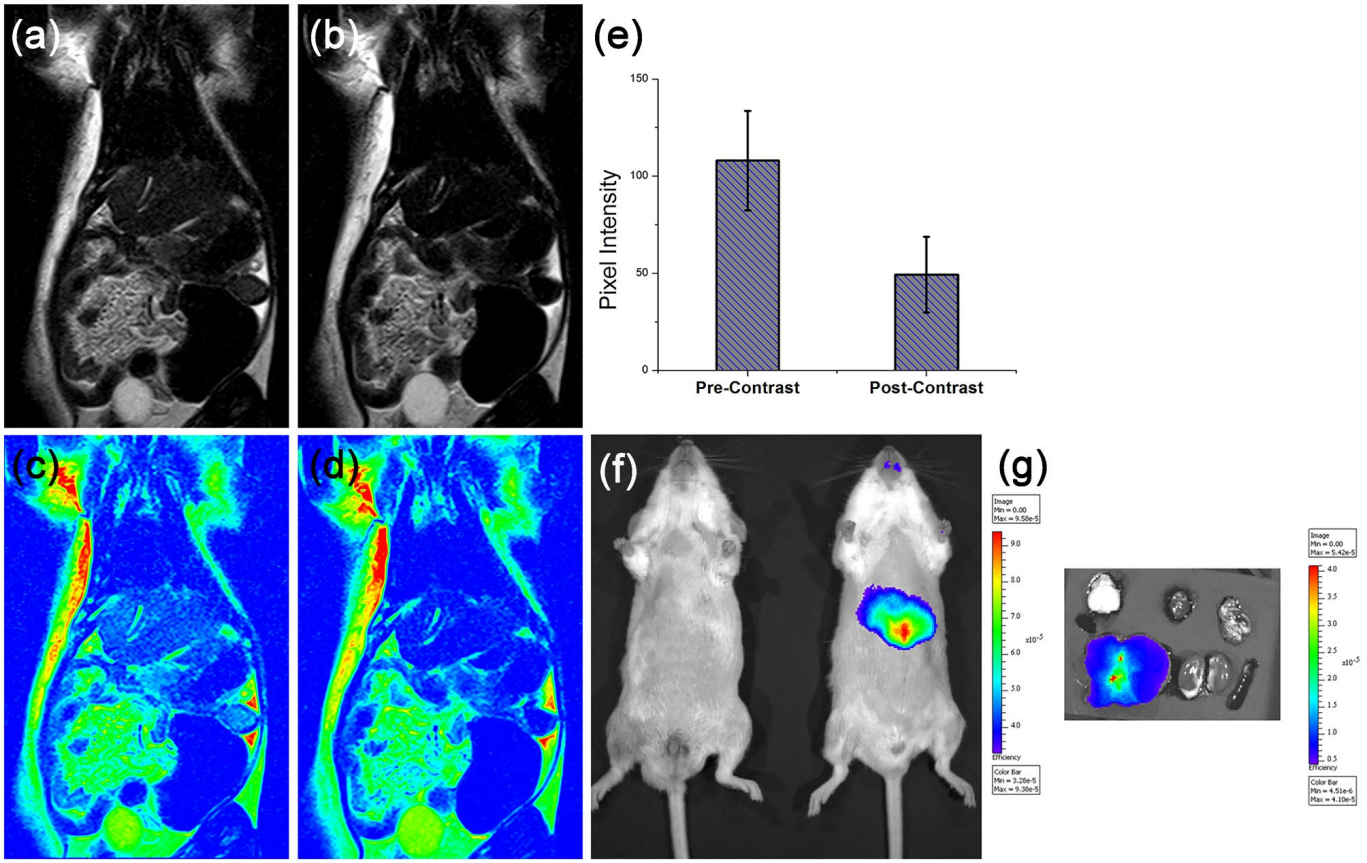
In vivo MR imaging using developed SNs. (**a** & **b**) Pre and Post ASPION injectected MRI images of liver fibrosis induced animal, respectively. (**c **& **d**) Corresponding pseudo colored images of pre and post contrast MRI for better visualization. (**e**) Signal intensity variation in percentage, extracted from liver area of pre and post contrast MRI. (**f**) In vivo fluorescence images of control and fibrosis induced mice acquired after administration of ASPION-AT intravenously. (**g**) Bio-distribution of ASPION-AT in different organs.

Before the MRI imaging, the animals were tested for the enhancement in liver specific enzymes, AST and ALT to confirm the liver fibrosis model development. Selected animals were anesthetized and subjected to MRI using a 1.5 T MRI scanner. Pre and post contrast T_2_ weighted coronal images and corresponding pseudo-colored images of the liver fibrosis induced animals are shown in Fig. 6a–d. ASPIONs enhance the image contrast of tissues through considerable shortening of T_2_ relaxation times. The shortening of T_2_ relaxation leads to a signal drop with a decrease in the pixel intensity at the sites where the SPIONs are accumulated. Average pixel intensity variation from the pre and post contrast liver also gave a remarkable difference of 54% between the two, indicating the clear visual difference between the images (p < 0.05), whereas for normal rats, liver contrast variation was only 14.49% after administering ASPIONs. The achievement of significant signal intensity variation before and after the contrast injection is desired by using the contrast material, for a clear visibility of the pathological condition for an accurate and easy diagnosis. This is met with the material developed under the current study (Fig. 6e).

The hypointensity observed in the post-contrast T_2_ weighted MR image is an indication of heavy uptake of ASPIONs by the liver cells. In the case ASPIONs, the carbohydrate polymer coating facilitates the enhanced permeability and retention based uptake of the material^52,53^. Within the hypointense liver, hyperintense streaks are visualized, which represents the Kupffer’s cell devoid fibrotic regions of the liver. There is an excessive accumulation of extracellular matrix in fibrosed liver and hence a decrease in the Kupffer’s cell density. As a result of the increase in the amount of collagen, the ratio of fibro-connective tissue verses liver cells increases at the fibrosed sites^17,54^.

### In vivo optical imaging using multimodal nanoprobe

The imaging efficiency of the developed multimodal nanoprobe (ASPION-AT) was evaluated in the mice model of liver fibrosis. ASPION-AT administered rodents were imaged after 15 min of intravenous administration. Elevated fluorescence signal intensity was observed from the sites corresponding to the fibrosed liver (efficiency = 7.92 × 10^–5^ ± 1.33 × 10^–5^) compared to the control mice kept aside during imaging, for comparison (Fig. 6f). The NIR emitting ASPION-AT reduced the interference from autofluorescence of the mice and the images can be very well distinguished without any further post processing^48^. The organs of each mouse were excised after 1 h to check the bio distribution of the nanoprobes, which showed high fluorescence intensity in the liver (Fig. 6g). Absence of the signal from other organs confirm the targeted delivery or specific uptake of ASPION-AT by the liver while injected through intravenous route.

### Histopathological analysis

**Figure 7:**
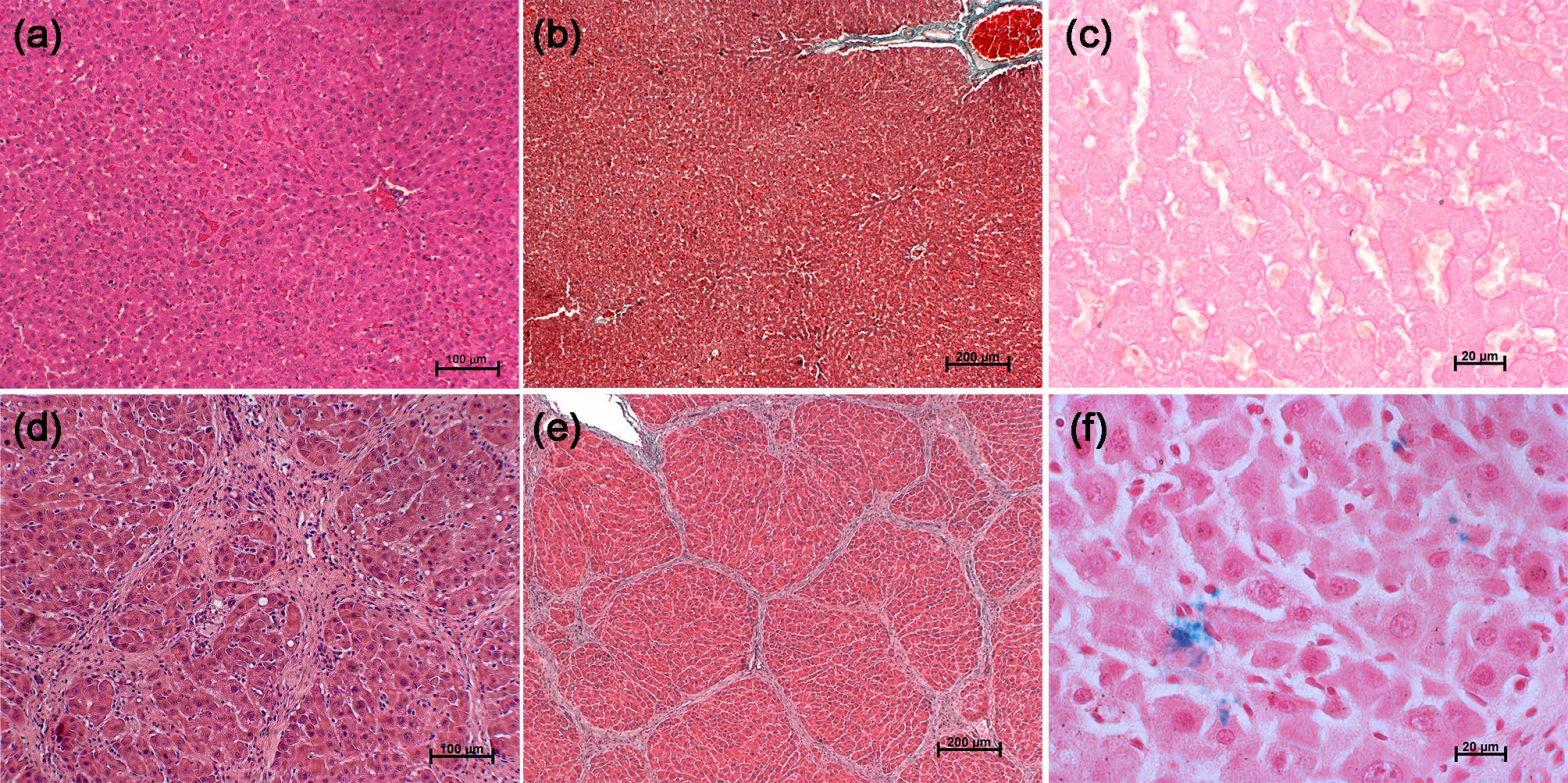
Histopathological evaluation of liver sections of control and fibrosis induced animals. Hematoxylin and Eosin, Masson’s Trichrome and Perl’s Prussian blue stained (**a**–**c**) control and (**d–f**) fibrosis induced liver sections.

H&E and MT stained images of normal liver revealed lobular architecture with central vein and radiating hepatic cords (Fig. 7a, b). However, the fibrotic liver revealed pronounced morphological alterations evidenced by disruption of the tissue architecture, moderate to severe necrosis of hepatocytes with infiltration of mononuclear cells and accumulation of fibers in peri-lobular and portal triad areas (Fig. 7d, e). The excessive accumulation of collagen fibers is very well distinguished in the MT stained sections of fibrosed liver (Fig. 7e), whereas this is absent in normal liver (Fig. 7b). Based on the H&E and MT staining procedures, the fibrous bridges dividing the liver into rounded islands of hepatic parenchyma resulting in the nodular formation surrounded by fibrous tissue has been observed in the liver. These histopathological observations reconfirm the development of fibrotic stage of liver in the rat model^29,55^. The iron uptake by the liver macrophages is insignificant in the case of PB stained normal liver (Fig. 7c) while the presence of iron in the fibrosed liver is confirmed, which was responsible for the hypointense MR image (Fig. 7f).

## Conclusions

In this study, Alginate stabilized iron oxide nanoparticles with a hydrodynamic diameter of 40 nm was successfully developed and evaluated as a potential candidate for in vivo MR imaging of liver fibrosis. ASPIONs possess very good cytocompatibility validating its applicability to use in living systems. ASPIONs showed a very good proton relaxivity ratio (r_2_/r_1_) of 30.53 at room temperature with an in vivo signal change of 54% in the T_2_ MRI after the intravenous administration. Application of varying magnetic field revealed the therapeutic efficiency of ASPIONs in hepatocellular carcinoma cells. Accounting these factors, ASPIONs can be considered as a potential candidate for the theranostics of liver associated diseases such as liver fibrosis. Further modification with NIR emitting Atto dye enabled the in vivo optical imaging with the enormous possibility of the probe to be used as a multifunctional theranostic tool. The developed SNs for in vivo imaging and hyperthermia of hepatocellular carcinoma and in vivo liver fibrosis is expected to play crucial role in the field of theranostics of liver diseases in the near future.

## Data Availability

The datasets generated and/or analysed during the current study are available from the corresponding author on reasonable request.
